# Evolution and phylogenetic characteristics of the first *Brucella canis* strain isolated from a human patient in Yunnan Province, China

**DOI:** 10.3389/fcimb.2025.1743711

**Published:** 2026-01-21

**Authors:** Qiuju Yang, Peng Wang, Xiangdong Yang, Su Zhao, Qing Zhang, Fuping Yang, Zhiguo Liu, Binbin Yu

**Affiliations:** 1 Yunnan Institute of Endemic Disease Control and Prevention, Yunnan Provincial Key Laboratory for Natural Focal Disease Control and Prevention, Kunming, China; 2 National Key Laboratory of Intelligent Tracking and Forecasting for Infectious Diseases, National Institute for Communicable Disease Control and Prevention, Chinese Center for Disease Control and Prevention, Beijing, China

**Keywords:** *Brucella canis*, brucellosis, pan-genome, phylogenetic analysis, whole-genome single nucleotide polymorphism

## Abstract

**Introduction:**

*Brucella canis* is a zoonotic pathogen that infects both dogs and humans, yet its evolutionary and phylogenetic characteristics are poorly understood.

**Methods:**

Here, we comprehensively characterized an isolated strain of *B. canis* through integrated bacteriological, comparative genomic, and whole-genome sequencing-based core genome single-nucleotide polymorphism (WGS-cgSNP) analyses.

**Results:**

*B. canis* YN20042 was isolated from a febrile patient (38 °C) with sweating and fatigue. The culture exhibited rough, grayish white, sticky, and opaque colonies. The isolate was identified as *Brucella* strain by a BCSP-31 polymerase chain reaction (PCR) assay, which yielded an amplicon of the expected 223-bp size, and was classified as a *B. canis* strain by conventional biotyping. The patient reported frequent contact with dogs and livestock. The strain showed a 99.99% average nucleotide identity to the *B. canis* reference strain ATCC 23365 (GCA_000018525.1). An *in silico* multilocus sequence typing (MLST) analysis showed that the strain belonged to sequence type 21, which was consistent with its classification within *B. canis*. The genome of strain YN20042 exhibited strong synteny with the reference strain and showed no detectable structural variations. It harbored 12 predicted virulence factors encompassing 71 associated genes, although it notably lacked the *wbpL* gene but contained a *Brucella suis mprF* gene. A further analysis identified predicted mutations in key virulence genes (*eryA*, *pagN*, *bmaC*, *cfa*1, and *cfa*2) and predicted multiple horizontally acquired genes, collectively suggesting a complex evolutionary trajectory involving both gene variants and potential recombination events. A WGS-SNP analysis revealed that YN20042 clustered closely with strains isolated from Zhejiang and Beijing, indicating a high degree of genetic relatedness.

**Conclusion:**

The first isolation of *B. canis* in the region expands the local spectrum of pathogenic *Brucella* and highlights the substantial infection risk for individuals with close dog and livestock contact. Enhanced surveillance, targeted screening of high-risk populations, and public health education are necessary to mitigate the risk of *B. canis* transmission.

## Introduction

*Brucella canis* is a pathogenic bacterium in dogs, with a zoonotic potential that has increased in recent years (Sánchez-Jiménez et al., 2015). *B. canis* is a significant public health threat to the canine breeding industry and to humans who have close contact with dogs (Liu et al., 2020). *B. canis* is zoonotic, similar to *Brucella abortus*, *Brucella suis*, and *Brucella mellitensis*, and handling or working with infected dogs can lead to human disease (Sebzda and Kauffman, 2023). Transmission to humans occurs through contact with infected canine material, most commonly abortion products (Lucero et al., 2010). In dogs, *B. canis* can cause reproductive failure, and in humans, it can cause fever, chills, malaise, and splenomegaly; however, it only infrequently causes human infections, likely due to its low virulence (Wallach et al., 2004). One study confirmed that *B. canis* isolated from urine indicated bacterial shedding, highlighting the potential role of urine in zoonotic transmission (Van Dijk et al., 2021). This evidence underscores how the pathogen can be introduced into non-endemic areas when infected dogs are imported from endemic regions, posing a threat to both the naive autochthonous dog population and humans. *B. canis* was isolated from South Dakota Indian reservations (USA), with a *B. canis* seroprevalence of 6.8% in clinical screening tests of 3,898 dogs over more than 4 years (Daly et al., 2020).

In China, *B. canis* strains were first isolated from imported beagles and domestic dogs in 1984 (Liu et al., 2020). A nationwide survey of *B. canis* infection was conducted in 23 provinces, with more than 100 *B. canis* isolates obtained from 20 provinces, all of which were obtained from dogs (Deqiu et al., 2002). Subsequently, only one case of *B. canis* infection was reported in a human patient in Zhejiang Province, China in 2011 (Piao et al., 2017). In 2019, *B. canis* CD3 and two other *B. canis* strains (WJ5 and YA4) were isolated from pet dogs in Sichuan, indicating a potential threat to public health posed by the subclinical infections in pet dogs (Yan et al., 2022). A multiple-locus variable-number tandem repeat analysis (MLVA) study of Chinese *B. canis* strains showed that MLVA-16 genotype 31 was detected in different provinces with over 20 years between outbreaks, with 5 of 10 Beijing *B. canis* outbreak isolates (in 2011) and the Jiangxi isolate (in 1987) belonging to MLVA-16 genotype 47, implying that *B. canis* strains may have spread throughout China (Di et al., 2014). The true prevalence of *B. canis* infections in humans across China remains undetermined. In this study, we report the first isolate of a *B. canis* strain from a patient in Yunnan Province. To investigate its origin and molecular phylogenetic characteristics, the isolate was characterized by combining a bacteriological examination, whole-genome sequencing-based single-nucleotide polymorphism (WGS-SNP) analysis, *in silico* multilocus sequence typing (MLST), and MLVA. These findings offer valuable insights for guiding public health surveillance, raising awareness of *B. canis* prevention, and improving early detection capabilities.

## Methods

### Sample source and species identification of strains

In this study, a *B. canis* strain, YN20042, was isolated from a febrile patient (highest temperature, 38 °C) in Yunnan Province, who presented with fatigue and profuse sweating. A 68-year-old female patient was admitted on 20 July 2020, with unexplained onset of dull abdominal pain exhibiting paroxysmal exacerbation, accompanied by abdominal distension and occasional cough with sputum production. An intermittent fever developed during the night of 31 July, with the highest temperature reaching 38 °C, accompanied by sweating and fatigue. This prompted pathogen screening of a blood culture. Procedures for isolating and culturing the strain followed standard bacteriological protocols (Jiang et al., 2020). Briefly, 5-mL blood samples were collected from the patient with suspected brucellosis, injected in a timely manner into a biphasic medium bottle in a biosafety cabinet, and incubated in an automated blood culture system (Qingdao Zhongchuang Huike Biotechnology Co., Ltd., HD-120). On 1 August, laboratory results confirmed that a *Brucella* strain had been detected. Suspected colonies were picked and preliminarily identified by BSCP-31 polymerase chain reaction (PCR), as previously reported (Baily et al., 1992). Briefly, the *Brucella* genus was detected by PCR using the primers BCSP31-B4-F (5′-TGGCTCGGTTGCCAATATCAA-3′) and BCSP31-B5-R (5′-CGCGCTTGCCTTTCAGGTCTG-3′). The reaction was performed in a 20-µL mixture containing 1.0 µL of template DNA, producing an amplicon of 223 bp, indicating positive results for *Brucella*. Subsequently, the strain was characterized by conventional biotyping following a standard protocol (Yagupsky et al., 2019), which included tests for growth features (CO_2_ and H_2_S), dye inhibition [basic fuchsin (BF) and threonine (TH)], agglutination with monospecific sera (A, M, and R), and phage lysis (Tb, BK2, and Wb).

### Serology and bacteriology screening of suspected cases

A total of 36 serum samples were collected for screening purposes, including blood specimens from 3 family members (the patient’s husband, son, and daughter-in-law) and 21 individuals engaged in animal husbandry from the same village. Additionally, five blood samples were obtained from sheep and seven were obtained from cattle (one of which was raised by the patient’s family). Rose Bengal plate tests (RBPTs) and standard tube agglutination tests (SATs) were performed on the collected serum, with the testing and interpretation performed according to standard approaches (Nielsen and Yu, 2010). Bacteriological screening of the 36 blood samples was conducted using standard methods. The patient’s epidemiological exposure history was also investigated.

### DNA preparation, whole-genome sequence assembly, and comparative analysis

Genomic DNA was extracted using a QIAamp DNA kit (Qiagen, Germany) based on the manufacturer’s instruction. The genomic DNA was then subjected to whole-genome sequencing. The sequencing library was prepared using a Nextera XT library preparation kit (Illumina Inc., San Diego, CA, USA), and sequencing was performed on an MGISEQ-2000 platform. The raw data were filtered and assembled using CLC Genomics Workbench V23.0.1 software with default parameters (QIAGEN, Hilden, Germany). The average nucleotide identity (ANI) of the strain was compared with five reference genomes [*B. abortus* (GCA_000369945.1), *B. melitensis* (GCA_000007125.1), *B. suis* (GCA_000007505.1), *B. ovis* (GCA_000016845.1), and *B. neotomae* (GCA_900446125.1)] using the Orthologous Average Nucleotide Identity Tool (Lee et al., 2016). With a threshold of >98% coverage and similarity to the reference sequence, virulence-associated genes were predicted by screening the assembled genomes against the Virulence Factor Database (VFDB) using ABRicate (Chen et al., 2005). Antimicrobial resistance genes were predicted using the Comprehensive Antibiotic Resistance Database (Alcock et al., 2020). A collinear analysis of each genome to the *B. canis* reference genome (GCA_000018525.1) was performed using Mauve software (Darling et al., 2004), and the core and unique genes of all 30 isolates (**Supplementary Table S1**) were analyzed using Panaroo tools (Tonkin-Hill et al., 2020). Variant calling was performed using Snippy v4.6.0, followed by functional annotation by SnpEff v4.3t (Cingolani et al., 2012) to classify SNPs and InDels (insertions–deletions) by impact level (high, moderate, low, and modifier). Basic Local Alignment Search Tool-based screening with a stringent similarity cutoff (>0.7) was used to identify potential recombination events. In addition, putative horizontally transferred genes (hGTs) were identified via HGTector (https://github.com/qiyunlab/HGTector) (Zhu et al., 2014).

### Genotyping, pangenome, and WGS-SNP analyses

The assembled genomes of the strain were subjected to *in silico* MLST, which produced nine locus allelic profiles, and *in silico* 16-locus MLVA typing (Al Dahouk et al., 2007) was performed using a purpose-written script (MLVA_finder) applied to genome assemblies, as described by Vergnaud et al. (2018). A worldwide MLVA of 83 *B. canis* strains (**Supplementary Table S1**) was performed to investigate the molecular relationships among the strains. A dendrogram was constructed using the unweighted pair group method with arithmetic mean (UPGMA) algorithm in BioNumerics 8.0 software. Furthermore, 23 *B. canis* strains (**Supplementary Table S2**) with close genetic relationships to YN20042 were selected to construct a dendrogram using the UPGMA method. In addition, an MLST comparative analysis of 514 *Brucella* strains (**Supplementary Table S3**) was conducted using Grapetree software (Zhou et al., 2018). All 29 *B. canis* genome sequences (**Supplementary Table S4**) were downloaded from GenBank, with *B. canis* ATCC 23365 (GCA_000018525.1) as the reference genome. The maximum-likelihood phylogenetic trees of the 30 *B. canis* strains were constructed using IQ-TREE with 1,000 bootstrap replicates. The resulting Newick tree file was imported into iTOL v6.5.7 (Letunic and Bork, 2021) for visualization.

## Results

### *Brucella canis* isolation and species identification

**Figure 1:**
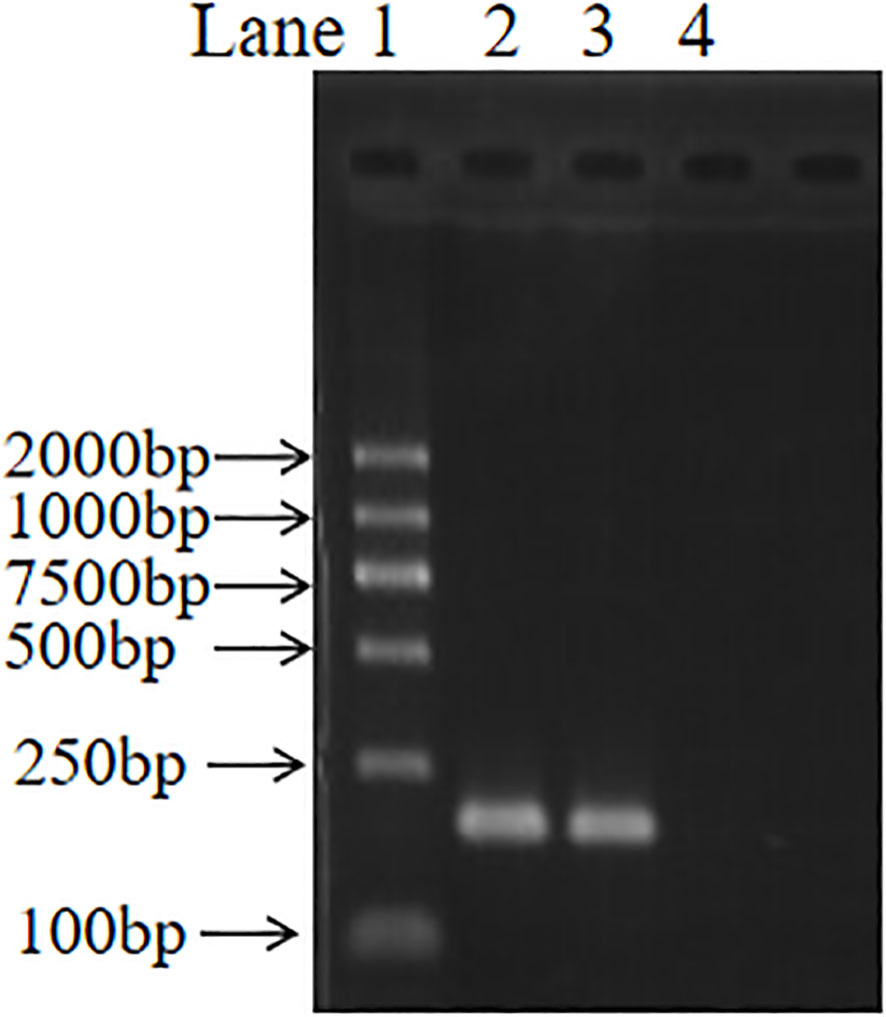
BCSP-31 PCR amplification of *Brucella canis* strain YN20042. Lanes: 1, DNA marker (DL2000); 2, positive control (*Brucella suis* S2 DNA); 3, test strain YN20042; 4, negative control (nuclease-free water). The expected amplicon size was 223 bp.

The blood culture results indicated the presence of a suspected *Brucella* species, and morphological observations revealed that the colonies were rough, grayish white, sticky, dry, and opaque. The strain was further identified by a BCSP-31 PCR assay, and a specific 223-bp size band was observed (**Figure 1**). The strain was determined to be a *Brucella* strain. Conventional biotyping results confirmed the isolate as *B. canis*, based on positive agglutination with R serum, growth in the presence of basic fuchsin, and resistance to lysis by Tb, BK^2^, and Wb phage (**Table 1**).

**Table 1:** Biotyping profile of the *B. canis* YN20042 strain.

Key	Strain	Growth feature				Agglutination with monospecific sera			Phage lysis test			No.	Interpretation
		CO_2_	H_2_S	BF	TH	A	M	R	Tb	BK_2_	Wb		
Reference strain	544	+	+	+	−	+	−	−	CL	CL	CL	1	*B. abortus* bv. 1
	16M	−	−	+	+	−	+	−	NL	CL	NL	1	*B. melitensis* bv. 1
	1330	−	++	−	+	+	−	−	NL	CL	CL	1	*B. suis* bv. 1
Tested strain	YN20042	−	−	+	−	−	−	+	NL	NL	NL	1	*B. canis*

BF, basic fuchsin; TH, threonine; CL, complete lysis; NL, no lysis; +, positive; −, negative.

### Serological testing and epidemiological investigation

The patient’s serum sample was positive on a rough antigen RBPT but negative on a standard RBPT. Conversely, it was negative on both rough and standard (smooth) SATs. An epidemiological survey found that the patient was frequently in contact with dogs and livestock (cattle and sheep). Furthermore, the serological and bacteriological screening results indicated that all 36 serum samples tested negative using both methods.

### Genome profile and comparative genome analysis

**Figure 2:**
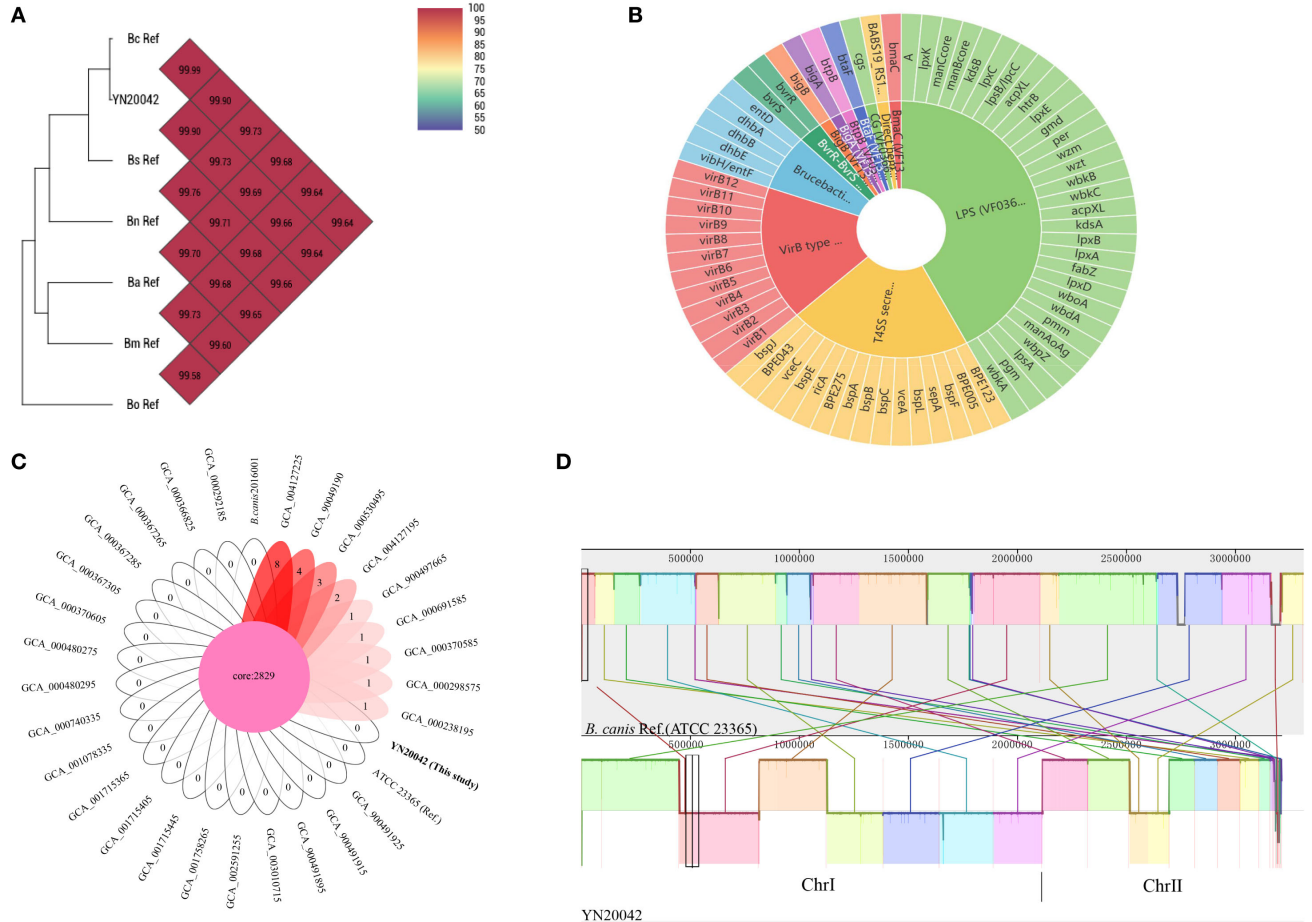
Comparative genomic analysis of the isolated strain included an average nucleotide identity (ANI) analysis **(A)**, prediction of virulence-associated genes **(B)**, core gene analysis **(C)**, and genome collinearity analysis **(D)**. **(A)** shows the ANI of YN20042 compared to five other classical *Brucella* sp*ecies*, including **(*B*)***abortus* (GCA_000369945.1) (Ba), *B. melitensis* (GCA_000007125.1) (Bm), *B. suis* (GCA_000007505.1) (Bs), *B. ovis* (GCA_000016845.1) (Bo), and *B. neotomae* (GCA_900446125.1) (Bn). The number in the figure indicates the ANI value between the two compared genomes. **(B)** illustrates the composition and distribution of virulence-associated genes in the isolated *B. canis* strain. A total of 30 genes were associated with lipopolysaccharide synthesis (LPS, VF0367; green), 15 with T4SS secreted effectors (VF0695; orange), 12 with the VirB type IV secretion system (red), 5 with brucebactin siderophore synthesis (VF0692; light blue), and 2 with the BvrR and BvrS regulatory system (VF0368; light green). The remaining seven virulence factors were each represented by a single gene (indicated by distinct colors). **(C)** displays the core and unique gene counts among the 31 *B. canis* strains, represented by a Venn diagram. The core gene set is shown in the central region, while the number of unique genes per strain is indicated in the respective lobes. **(D)** illustrates the genomic collinearity between the *B. canis* reference genome and strain YN20042. Homologous regions are indicated by similarly colored blocks, with connecting lines highlighting syntenic gene sequences.

The genome assembly showed a genome size of 3,214,139 bp, with 31 scaffolds. The N50 was 250,155 bp, and the N75 was 190,618 bp. The length of the largest scaffold was 368,314 bp, the total length of the coding genes was 2,785,311 bp, and the GC content was 55.11%. The ANI to the *B. canis* ATCC 23365 (GCA_000018525.1) genome was 99.99% (**Figure 2A**), implying that the strain was *B. canis*. The analysis predicted 12 virulence factors encompassing 71 associated genes (**Figure 2B**; **Table 2**). However, the *wbpL* gene was absent. The *Brucella suis mprF* gene was identified. Compared with the smooth strain, the *wbpL* gene was absent. *wbpL* encodes an undecaprenyl-phosphate alpha-N-acetylglucosaminyltransferase, which is important in the O-antigen synthesis of lipopolysaccharide (LPS) in smooth *Brucella*. A core gene analysis revealed that all *B. canis* strains shared 2,829 core genes. The strain isolated in the present study did not contain any unique genes, while the number of unique genes in the other nine strains ranged from one to eight (**Figure 2C**). This high degree of genomic conservation underscores the remarkable genetic stability of *B. canis*. A genome collinearity analysis revealed that all strains exhibited a high degree of synteny with the *B. canis* reference genome, with most genomic regions aligning closely to the reference (**Figure 2D**). This strong collinearity reflected a highly conserved genome structure and suggested the absence of large-scale genomic structural variations.

**Table 2:** The composition of virulence-associated genes and antimicrobial resistance genes in the *B. canis* YN20042 strain.

Virulence factor	Gene count	Virulence gene name	Predicted biology function
LPS (VF0367)	30	*A*, *lpxK*, *manBcore*, *manCcore*, *kdsB*, *lpxC*, *lpsB/lpcC*, *acpXL*, *htrB*, *lpxE*, *gmd*, *per*, *wzm*, *wzt*, *wbkB*, *wbkC*, *acpXL*, *kdsA*, *lpxB*, *lpxA*, *fabZ*, *lpxD*, *wbdA*, *wboA*, *pmm*, *manAoAg*, *wbpZ*, *lpsA*, *pgm*, and *wbkA*	Immune modulation
T4SS secreted effectors (VF0695)	15	*BPE123*, *BPE005*, *BPE043*, *BPE275*, *bspF*, *sepA*, *bspL*, *vceA*, *bspC*, *bspA*, *bspB*, *ricA*, *bspE*, *vceC*, and *bspJ*	Effector delivery system
VirB type IV secretion system (VF0365)	12	*virB1*, *virB2*, *virB3*, *virB4*, *virB5*, *virB6*, *virB7*, *virB8*, *virB9*, *virB10*, *virB11*, and *virB12*	Effector delivery system
Brucebactin (VF0692)	5	*vibH/entF*, *dhbE*, *dhbB*, *dhbA*, and *entD*	Nutritional or metabolic factor
BvrR-BvrS (VF0368)	2	*bvrS* and *bvrR*	Regulation
Direct heme uptake system (VF0693)	1	*BABS19_RS15905*	Nutritional or metabolic factor
BmaC (VF1341)	1	*bmaC*	Adherence
BigB (VF1345)	1	*bigB*	Adherence
BigA (VF1344)	1	*BigA*	Adherence
CG (VF0366)	1	*cgs*	Immune modulation
BtpB (VF0522)	1	*btpB*	Immune modulation
BtaF (VF1343)	1	*btaF*	Adherence
Antimicrobial resistance genes	1	*Brucella suis mprF*	Defensin resistance and antibiotic target alteration

### Point mutations, genetic recombination, and horizontal gene transfer of *B. canis*

**Figure 3:**
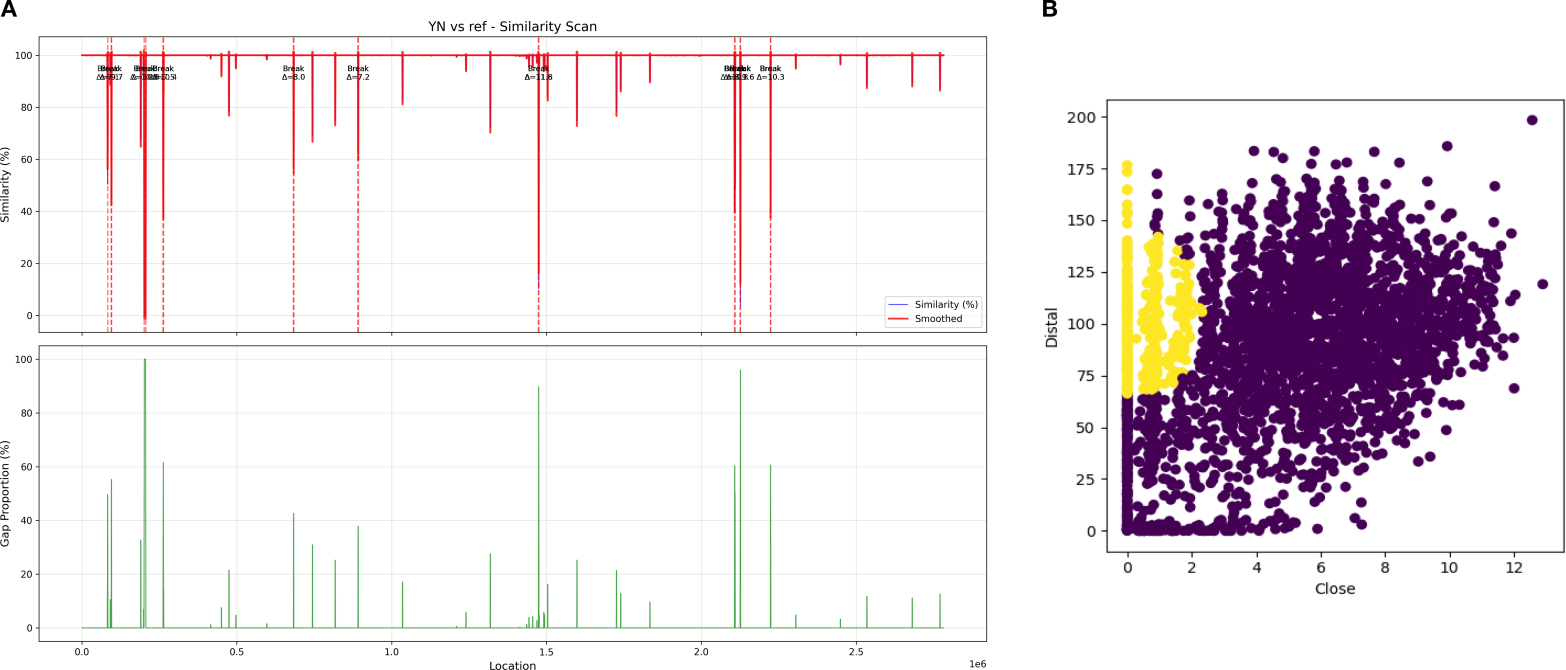
**(A)** A similarity scan of strain YN20042 (YN) compared to a reference sequence. Note: The top panel shows the percent similarity curve, and the bottom panel shows the genome region coverage. The graph displays the percent similarity (blue line) between the YN sequence and the reference sequence across genomic positions and its smoothed trend (orange line) (*x*-axis, unit: 1×10^6^ bp). The lower bar plot indicates sequence coverage in the corresponding regions. Δ indicates the similarity value in this breakpoint. **(B)** Scatter plot analysis of horizontal gene transfer (HGT) predictions. Note: The scatter plot compares the distal weight against the proximal weight of individual genes, with each dot representing one gene. The distribution reveals distinct clustering patterns, which aids in the identification of potential horizontal gene transfer events. Genes predicted to be acquired through HGT are highlighted (yellow dot), illustrating their separation from the core genomic background based on weight distribution in distal versus proximal genomic contexts.

The spectrum of high-impact mutations identified in the *B. canis* YN20042 strain that encompassed frameshift, stop-gain, stop-loss, and splice variants converged to disrupt two fundamental pillars of *B. canis* pathogenicity (**Table 3**). First, the mutations dismantled the core machinery for host invasion and intracellular survival by ablating erythritol metabolism (*eryA*), iron acquisition (*pagN*), adhesion (*bmaC*), and membrane integrity (*cfa* 1 and *cfa* 2). Second, they crippled the pathogen’s resilience by disabling critical stress response (*ppk* 1 and *hslU* 2) and metabolic flexibility (*edd*, *puuA*, *puuD*, and *araB* 1) networks, collectively rendering the strain avirulent. The recombination analysis revealed no evidence of segmental recombination events. Although a breakpoint scan identified multiple loci with sharp similarity transitions (Δ similarity: 7.15–11.79) indicating potential recombination (**Figure 3A**), the analysis revealed the genome-wide similarity distribution and local variations between the YN20042 and reference sequences. However, these findings remain preliminary; further investigation is required to confirm the nature and role of these genomic features. A horizontal gene transfer analysis identified multiple potential recombination events and predicted over 60 horizontally acquired genes in strain YN20042 (similarity > 0.7) (**Figure 3B**). The specific adaptive functions of these acquired genes and their effects on strain fitness, however, require further experimental validation.

**Table 3:** Genome SNP variant analysis results of the *B. canis* YN20042 strain.

Gene	Variant consequence	Predicted biological impact on gene function
*Cfa* 1	Stop loss and splice region variant	Disruption of cell membrane adaptation (cyclopropane fatty acid synthase activity impaired)
*pagN*	Stop gain (nonsense)	Loss of iron acquisition (outer membrane iron uptake protein function abolished)
*hslU* 2	Stop loss and splice region variant	Impaired protein quality control and stress response (ATP-dependent protease subunit inactivated)
*bmaC*	Stop loss and splice region variant	Compromised adhesion (putative autotransporter/adhesin function disrupted)
*Cfa* 2	Frameshift variant	Similar to cfa_1: loss of cyclopropane fatty acid synthase function affecting membrane adaptation
*Ppk* 1	Frameshift variant	Disrupted stress response and persistence (polyphosphate synthesis abolished)
*thpA*	Frameshift variant	Defective thymidine catabolism and perturbed nucleotide salvage
*araB* 1	Frameshift variant	Blocked arabinose utilization pathway (ribulokinase inactivated)
*puuA*	Frameshift variant	Disrupted nitrogen metabolism (putrescine degradation pathway impaired)
*Edd*	Frameshift variant	Impaired nucleotide salvage pathway (thymidine catabolism disrupted)
*eryA*	Frameshift variant	Severe attenuation of virulence (erythritol kinase function abolished, preventing utilization of a key carbon source)
*puuD*	Frameshift variant	Further disruption of nitrogen metabolism (later step in the putrescine degradation pathway)

### Genotyping and phylogeny analysis of strain YN20042

**Figure 4:**
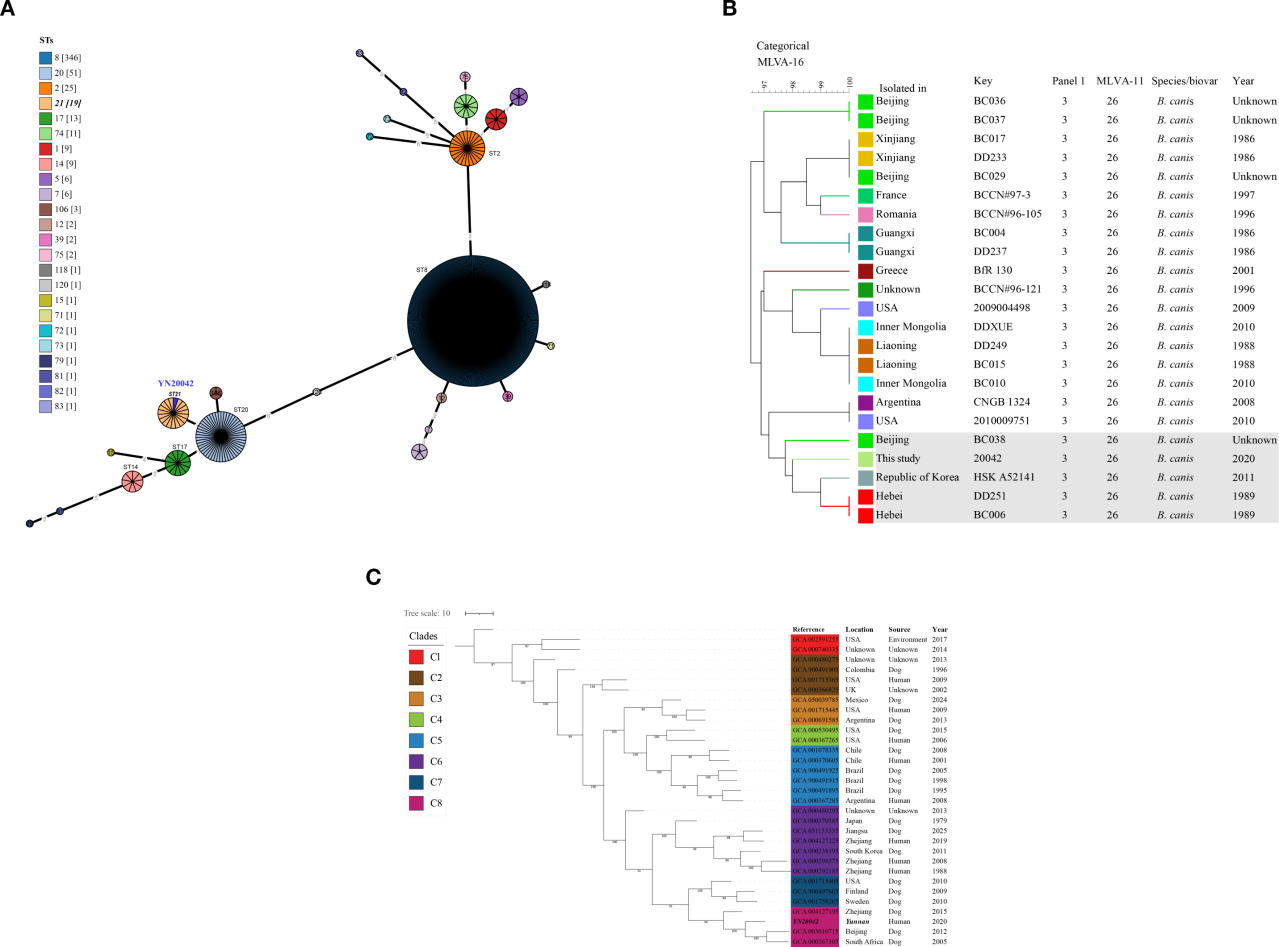
**(A)** Population structure of *B*. *canis* strains from this study and from various geographic regions, with sequence types distinguished by color. Numbers in parentheses indicate the count of strains per ST, with the strain from this study (YN20042) marked with dark blue. **(B)** The MLVA-16 dendrograms of 23 *B*. *canis* strains based on the unweighted pair group method with arithmetic mean. **(C)** WGS-cgSNP phylogenetic analysis of 31 *B*. *canis* strains worldwide based on the maximum-likelihood phylogenies algorithm with 1,000 bootstrap replicates. The *B*. *canis* strain in this study is marked with black italics, and the numbers on the nodes represent bootstrap values.

An *in silico* MLST analysis identified the strain as sequence type 21 (ST21), confirming its placement within the *B. canis* population (**Figure 4A**). MLVA-11 genotyping further assigned panel 1 and the complete MLVA-11 genotype as 3 and 26, respectively. A genetic relationship comparison using the MLVA-16 profiles indicated that the strain clustered closely with *B. canis* isolates from Beijing, Hebei, and the Republic of Korea (**Figure 4B**). In addition, a WGS-SNP-based phylogenetic tree constructed from the 31 global *B. canis* strains revealed eight major clades (**Figure 4C**). The strain in this study was evolutionarily closest to strains originating from Zhejiang and Beijing, China.

## Discussion

In this study, a comprehensive molecular investigation was performed on a *B. canis* strain isolated from a patient in Yunnan who exhibited an apparent clinical syndrome. In the present case, the patient did not follow targeted protective measures while breeding dogs and other livestock (cattle and sheep), and did not use disinfectants while cleaning the livestock pens. Therefore, the patient was considered to be potentially infected with *B. canis*, caused by direct or indirect contact with dogs and livestock. In humans, *B. canis* can be the source of chronic debilitating symptoms characteristic of this genus, such as undulant fever, splenomegaly, and lymphadenopathy (Djokic et al., 2023). In this study, a negative serological test (SAT) result highlighted the deficiencies in the current diagnostic methods for detecting *B. canis* infection. Therefore, the use of highly sensitive and specific methods, such as enzyme-linked immunosorbent assays and immunofluorescence assays, is recommended for the diagnosis of *B. canis* brucellosis.

The *wbpL* gene, which is necessary for LPS synthesis (Barbieux et al., 2024), was absent in this strain. Moreover, significant differences in *wbkF* and *wbkD* genes have been observed between smooth and rough *Brucella* species (Zygmunt et al., 2009). The strain has a repertoire of 71 virulence-associated genes, spanning essential functional modules that include immune modulation (LPS pathway), adhesion (*bmaC* and *bigA/B*), effector delivery (T4SS and secreted effectors), and nutritional acquisition systems, suggesting a largely preserved virulence architecture with potential functional compensation (Anbazhagan et al., 2024). Although mutations in several key genes, such as *eryA* and *pagN*, are predicted to impair specific functions related to host invasion and intracellular survival, the overall pathogenic effects of these genetic alterations, particularly against the background of an otherwise intact virulence repertoire, remain to be comprehensively evaluated through integrated phenotypic and functional studies.

The observed genomic features of YN20042 *B. canis*, including notable evolutionary conservation, a highly restricted accessory genome, and strong collinearity with the reference genome, collectively underscore its remarkable genetic stability, which likely contributes to its adaptive persistence within host populations (Suárez-Esquivel et al., 2020). Horizontal gene transfer has made unique contributions to the *Brucella* genome evolution, even within its typically intracellular lifestyle (Wattam et al., 2009). The observed localized recombination signatures of the YN20042 genome when aligned with previous genomic studies revealed that *Brucella* has an open pangenome with abundant accessory genes, based on the identification of hGTs from 261 families across 255 genomes (Yang et al., 2024).

Historically, *B. canis* strains were epidemic in southern regions of China, such as Guangxi and Zhejiang provinces (Deqiu et al., 2002). The isolated location of the strain in this study was adjacent to Guangxi; however, the molecular correlation among strains needs further investigation. A WGS-SNP phylogenetic analysis revealed that the strain isolated in this study exhibited high genetic similarity to *B. canis* isolates from Zhejiang, Beijing, and South Africa. This finding suggested that sporadic cases likely originated from closely related *B. canis* lineage, underscoring the importance of enhancing *B. canis* surveillance. The observed domestic strain similarity may be associated with China’s growing pet dog population and internal trade, with 50.08 million dogs owned nationally as of 2018 (Seongjin et al., 2020). Between 2012 and 2013, 38 suspected brucellosis cases in pet dogs from Beijing were tested; 18 were seropositive, indicating an infection rate of 47.37% (18/38); and *Brucella* spp. were isolated from five of these seropositive dogs, four of which were identified as *B. canis* (Wang et al., 2014). This finding indicates the active circulation of *B. canis* within the urban pet dog population. Further investigation is warranted to elucidate the potential role of interprovincial trade and movement of livestock and dogs in facilitating pathogen dispersal and accurately characterize the actual transmission patterns of *B. canis* both within the region and across China. The observed genetic similarity to the South African strain suggests plausible links to international dogs or livestock trade. However, conclusive evidence tracing the transmission route requires further investigation. For example, *B. canis* was first detected in aborted fetuses in Italy, and genomic analyses identified the strains as ST21. However, in a broader scale comparison using publicly available genomes, the closest genome, isolated in China, differed by more than 50 alleles; thus, it is essentially impossible to determine the likely origin of a cluster epidemic (De Massis et al., 2021). Therefore, enhancing *B. canis* strain surveillance based on genome sequencing will provide more clues for understanding the evolution and transmission of *B. canis* on both regional and national scales. This study underscored that *B. canis* remains a significant threat to public health as people continue to have close contact with dogs and livestock. The public needs to be more aware of *B. canis* transmission and preventive measures.

The present study provides valuable insights into the evolution and phylogenetics of a *B. canis* strain from Yunnan; however, several limitations should be acknowledged. The limited local sample size, combined with the absence of positive results in livestock, prevents definitive conclusions regarding a local infection source. Furthermore, the lack of national-scale surveillance data restricts meaningful molecular epidemiological comparisons. Implementing routine *B. canis* screening in livestock and dogs is therefore recommended to enhance surveillance.

## Conclusion

In this study, we reported the isolation and genomic characterization of *B. canis* strain YN20042 from a febrile patient in Yunnan, China. A whole-genome analysis demonstrated a highly conserved and structurally stable genomic architecture (ST21), with functionally relevant mutations in key virulence determinants (*eryA*, *pagN*, *bmaC*, *cfa* 1, and *cfa* 2) and signatures indicating horizontal gene transfer of many acquired genes, suggesting an evolutionary history shaped by both gene loss and recombination. A phylogenetic analysis revealed that YN20042 clustered within a clade containing strains from Zhejiang, Beijing, and South Africa, supporting the circulation of a shared lineage associated with sporadic human infections. Together, these results reinforced *B. canis* as an ongoing yet underrecognized public health concern, particularly in populations with occupational or companion animal contact, and underscored the importance of integrated surveillance and targeted research to characterize transmission pathways at the human–animal interface.

## Data Availability

The datasets presented in this study can be found in online repositories. The names of the repository/repositories and accession number(s) can be found in the article/**Supplementary Material**.
